# Network-Based Differences in Top–Down Multisensory Integration between Adult ADHD and Healthy Controls—A Diffusion MRI Study

**DOI:** 10.3390/brainsci13030388

**Published:** 2023-02-23

**Authors:** Marcel Schulze, Behrem Aslan, Ezequiel Farrher, Farida Grinberg, Nadim Shah, Markus Schirmer, Alexander Radbruch, Tony Stöcker, Silke Lux, Alexandra Philipsen

**Affiliations:** 1 Department of Psychiatry and Psychotherapy, University of Bonn, 53113 Bonn, Germany; 2 Faculty of Psychology and Sports Science, Bielefeld University, 33615 Bielefeld, Germany; 3 Institute of Neuroscience and Medicine 4, INM-4, Forschungszentrum Jülich, 52425 Jülich, Germany; 4 Department of Neurology, RWTH Aachen University, 50264 Aachen, Germany; 5 JARA-BRAIN-Translational Medicine, 52056 Aachen, Germany; 6 Institute of Neuroscience and Medicine 11, INM–11, JARA, Forschungszentrum Jülich, 52425 Jülich, Germany; 7 Clinic for Neuroradiology, University Hospital Bonn, 53127 Bonn, Germany; 8 J. Philip Kistler Stroke Research Center, Massachusetts General Hospital, Harvard Medical School, Boston, MA 02115, USA; 9 German Center for Neurodegenerative Diseases (DZNE), 53127 Bonn, Germany

**Keywords:** ADHD, multisensory integration, top–down, structural connectome

## Abstract

Background: Attention-deficit–hyperactivity disorder (ADHD) is a neurodevelopmental disorder neurobiologically conceptualized as a network disorder in white and gray matter. A relatively new branch in ADHD research is sensory processing. Here, altered sensory processing i.e., sensory hypersensitivity, is reported, especially in the auditory domain. However, our perception is driven by a complex interplay across different sensory modalities. Our brain is specialized in binding those different sensory modalities to a unified percept—a process called multisensory integration (MI) that is mediated through fronto-temporal and fronto-parietal networks. MI has been recently described to be impaired for complex stimuli in adult patients with ADHD. The current study relates MI in adult ADHD with diffusion-weighted imaging. Connectome-based and graph-theoretic analysis was applied to investigate a possible relationship between the ability to integrate multimodal input and network-based ADHD pathophysiology. Methods: Multishell, high-angular resolution diffusion-weighted imaging was performed on twenty-five patients with ADHD (six females, age: 30.08 (SD: 9.3) years) and twenty-four healthy controls (nine females; age: 26.88 (SD: 6.3) years). Structural connectome was created and graph theory was applied to investigate ADHD pathophysiology. Additionally, MI scores, i.e., the percentage of successful multisensory integration derived from the McGurk paradigm, were groupwise correlated with the structural connectome. Results: Structural connectivity was elevated in patients with ADHD in network hubs mirroring altered default-mode network activity typically reported for patients with ADHD. Compared to controls, MI was associated with higher connectivity in ADHD between Heschl’s gyrus and auditory parabelt regions along with altered fronto-temporal network integrity. Conclusion: Alterations in structural network integrity in adult ADHD can be extended to multisensory behavior. MI and the respective network integration in ADHD might represent the maturational cortical delay that extends to adulthood with respect to sensory processing.

## 1. Introduction

Attention-deficit/hyperactivity disorder (ADHD) is a neurodevelopmental disorder with core symptoms of inattention, hyperactivity, and/or impulsivity [1]. Over the past years, ADHD was not only considered as a childhood disorder, since it extends into adulthood in 40–50% of patients [2]. Strongly connected to the core symptoms, ADHD is considered to be an executive function-disorder with most evidenced difficulties in the domains of working memory and inhibition [3]. Moreover, emerging evidence points to the existence of sensory-processing deficits. Although research regarding sensory processing in ADHD is rather limited, is has been shown that children with ADHD exhibit dysfunctional sensory processing nearly across all sensory modalities [4]. In adults, sensory processing seems to be normalized for most of the modalities, although studies reported lower stimulus discrimination thresholds for the visual and auditory modality [5]. Especially, the auditory modality seems to be associated with dysfunctional processing since early stimulus modulatory components are reported to be deviant. Those early component dysfunctions could be associated with a higher auditory distractibility (e.g., the inability to ignore background noise) at a behavioral level [6,7]. In reality, we are bombarded with parallel stimuli across the different sensory modalities; hence, our perception is the result of a sensitive interplay across our senses. To obtain a coherent perception, our brain combines different sensory modalities–a process that is called multisensory integration (MI) [8]. Only a few studies have investigated MI in ADHD, reporting mixed results [9,10,11,12]. Overall, these studies can be summarized with regard to the stimuli quality employed. When applied simple stimuli, e.g., a simple visual flash or an auditory beep-tone, patients with ADHD integrate audiovisual inputs to a similar degree compared to healthy controls. However, when confronted with complex audiovisual input, e.g., speech, patients with ADHD showed disrupted MI. Those findings can be explained in light of the underlying concepts of MI. Adjusting gain and stimulus saliency for simple stimuli is driven by automatic bottom–up attention moderated by primary sensory areas. In contrast, complex stimuli need further adjusting from higher association areas, i.e., top–down attention from frontal regions [13]. Structural connectivity as an index of network integrity in ADHD gained more scientific attention during the past years. Disturbed connectivity in ADHD was reported in the default-mode network (DMN), an intrinsic, spontaneous activation of brain areas at rest, usually suppressed in the presence of a task [14], limbic networks, visual attention networks, and fronto-temporal and fronto-parietal networks [15,16,17]. Following through on previous work completed by the authors, we hereby have the opportunity to explore MI in relationship to white matter (WM) structural connectivity in adult ADHD. As reported in Schulze et al. 2022 [12], we applied a classical MI paradigm: the McGurk illusion (MCG) [18]. In brief, incongruently presented audio–visual speech-phonemes resulted, in the case of successful integration, to a new, fused percept other than from the visual- and auditory-presented ones. Phonemes were presented across different conditions (120 trials each): unimodal auditory, unimodal visual, bimodal-congruent, and bimodal-incongruent datasets [19]. Importantly, for the bimodal-incongruent condition, ADHD participants showed significantly less MI, as they reported the auditory phoneme more often rather than as a fused percept. In the current work, we pose the question whether a disturbed MI process is related to structural–white matter connectivity in adult ADHD. More specifically, since there are overlaps in those regions that are associated with polymodal sensory processing (e.g., insula and temporal cortices) and ADHD pathophysiology, we assume altered network integrity in networks associated with early sensory processing and polymodal, sensory binding areas in adult ADHD.

## 2. Materials and Methods

### 2.1. Participants

As described in our previous work [12], we recruited 25 patients with ADHD (6 f, mean age: 30.08 (SD:9.3)) via the psychiatric outpatient department. The psychiatric sample was compared to 24 healthy controls (9 females; age: 26.88 (SD: 6.3) years). Patients with ADHD received a standardized diagnosis according to Diagnostic and Statistical Manual of Mental Disorders [20,21,22]. In case of medication with stimulants, the patients were asked to discontinue at least 24 h prior to the study. The full screening procedure is described in [11]. Applied questionnaires to further assess ADHD symptoms were the Conners Adult ADHD rating scales (CAARS) long version self-rated [23]. For the retrospective assessment of ADHD symptom in childhood, we used the Wender Utah Rating Scale (WURS-k) [24].

### 2.2. MRI Protocol

MR images were acquired on a 3 T MRI scanner (Magnetom Skyra, Siemens Healthineers) using a 32-channel head coil for signal reception. Magnetization-prepared rapid gradient echo (MP-RAGE) T1-weighted images were acquired with an acquisition time of 2 min 40 s using controlled aliasing in parallel imaging results in higher acceleration (CAIPIRINHA) and elliptical sampling (repetition time (TR) = 2500 ms, echo time (TE) = 3.55 ms, inversion time = 1100 ms, flip angle = 7°, matrix size = 256 × 256 × 176, voxel size = 1.0 × 1.0 × 1.0 mm^3^, sagittal slice orientation, slice-parallel imaging acceleration factor 3, CAIPI shift 1, Turbofactor 192) [25,26]. Multishell high-angular resolution diffusion-weighted MRI (DWI) was performed with a simultaneous multislice (SMS) Spin-Echo EPI sequence employing threefold slice-acceleration [27]. The protocol parameters were: TR = 5200 ms; TE = 106 ms; b-values (gradient-encoding directions) = 0 (7), 1000 (30), 2000 (40), 3000 (50) s/mm^2^; voxel size = 2.0 × 2.0 × 2.0 mm^3^; matrix size = 104 × 104 × 72 acquisition time = 11:26 min. In addition, five non-DW images were collected with reversed phase-encoding blips for the purpose of correcting susceptibility-induced distortions.

### 2.3. Diffusion MRI Data Analysis

The software package MRtrix3 was used to perform fiber tractography based on the multishell, multitissue constrained spherical deconvolution approach (MSMT-CSD) [28] via the following steps: (i) data denoising using the function dwidenoise [29] and Gibbs ringing artifacts [30]; (ii) correction of eddy current and susceptibility-induced distortions as well as motion using the FSL functions topup and eddy [31,32]; (iii) correction of B1 field heterogeneity using the ANTs function dwibiascorrect implemented in MRtrix3 [33]; (iv) estimation of the response function using the script dwi2response based on the “dhollander” algorithm [34]; (v) voxelwise estimation of the fiber-orientation distribution function using the MSMT-CSD method with the help of the function dwi2fod using the previously estimated response functions [35]; (vi) normalization of the fiber-orientation distribution across subjects and all tissue compartments (i.e., white/gray matter and cerebrospinal fluid); (vii) tissue segmentation of the T1-weighted image using the functions BET, FAST, and FIRST, available in FSL; (viii) registration of the DWI data to the segmented image using a rigid body registration algorithm with 6 degrees of freedom with the help of the function FLIRT, available in FSL; (ix ) segmentation and parcellation of the anatomical image in 84 cortical and subcortical regions using the Desikan–Killiany atlas as it is implemented in Freesurfer (https://surfer.nmr.mgh.harvard.edu/); (x) whole-brain anatomically constrained tractography (ACT) [36] using the function tckgen; (xi) filtering of these tractograms using the approach spherical-deconvolution informed filtering of tractograms (SIFT) [37] with the help of the function tcksift2; (xii) creation of the structural connectome by mapping the filtered streamlines to the parcellation image resulting in an 84 × 84 connectivity matrix for each individual using the function tck2connectome.

Statistically, group comparisons were performed at the edge-level using nonparametric permutation testing (n = 5000). Here, threshold-free network-based statistics were applied with familywise error-corrected *p*-values [38]. In a second step, MCG-fused scores (i.e., the percentage score of MI of the McGurk paradigm) were groupwise correlated with the connectome matrix to investigate a possible association of MI and structural connectivity.

### 2.4. Graph Theory

Local and global measures of brain networks can be analyzed with respect to graph theory. Here, a brain network is represented as a graph with its respective number of nodes and edges [39]. Implemented in the GRETNA toolbox [40], the following network metrics were calculated: global efficiency (i.e., efficiency of information transfer throughout the global nodal network), nodal local efficiency (i.e., efficiency of information transfer between neighboring nodes), shortest path length (i.e., least number of between-nodal connections), degree centrality (i.e., number of shortest paths that pass through as a bridging index between nodes), and clustering coefficient (i.e., quantification of nodal neighboring connectivity strength) [39,41]. Each global and local measure was statistically addressed using a false discovery rate (FDR)-corrected (*p* = 0.005) two-tailed t-test between ADHD and controls.

## 3. Results

### 3.1. Demographic Variables

For a complete sample description, please refer to our previous publication (Schulze et al., 2021). There was no difference in terms of age and gender.

### 3.2. McGurk Audiovisual Integration

**Figure 1:**
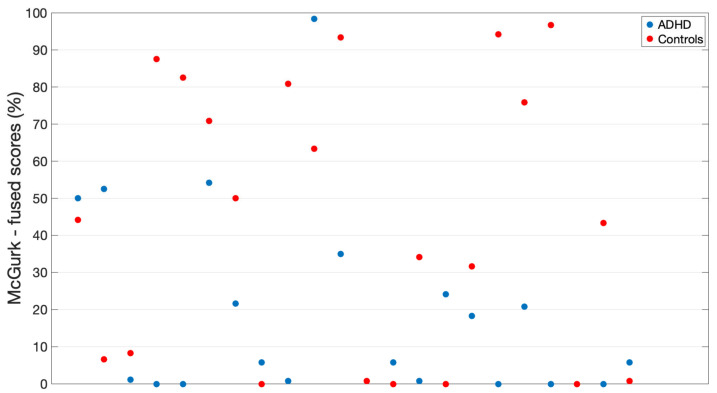
McGurk performance: percentage of successful fused integration.

The Mann–Whitney U test indicated that patients with ADHD integrated significantly less compared to healthy controls (Mean_ADHD_ = 18.01% (SD = 2.5), Mean_Controls_ = 45.9% (SD = 3.7), U= 160.5, *p* = 0.002; Figure 1).

### 3.3. Network-Based WM Connectivity

#### 3.3.1. ADHD > Controls

**Figure 2:**
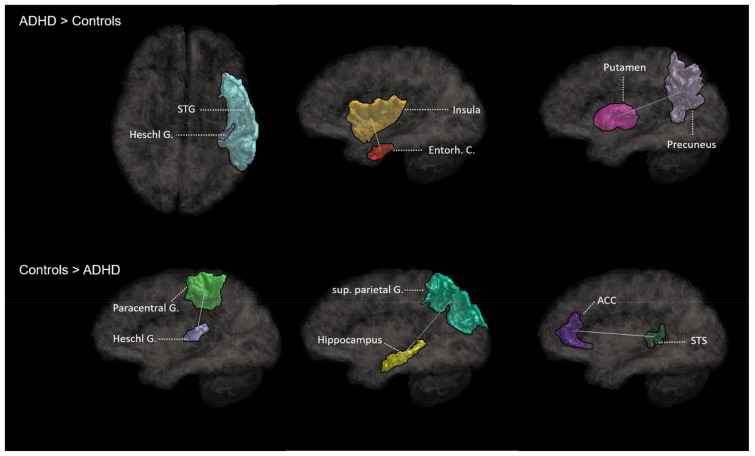
Hyper- (**top**) and hypo- (**bottom**) network connectivity of patients with ADHD compared to healthy controls. Abbreviations: STG, superior temporal gyrus; G, gyrus; Entorh. C., entorhinal cortex; sup. parietal G., superior parietal gyrus; ACC, anterior cingulate cortex; STS, superior temporal gyrus.

Higher network connectivity (FWE corrected *p* = 0.05) in ADHD compared to controls was found in the following networks: right entorhinal cortex and right insula, left entorhinal cortex and right cerebellum, right superiortemporal cortex and left Heschl gyrus, right putamen and right precuneus, and left parsopercularis and right precuneus. Detailed network information can be found in Table 1 and Figure 2.

#### 3.3.2. ADHD < Controls

Compared to patients with ADHD, higher network connectivity was revealed for healthy controls in the following networks: right anterior cingulum and right superior temporal sulcus, right cuneus and left lingual gyrus, right insula and right parsorbitalis, left nucleus accumbens and left paracentral gyrus, right paracentral gyrus and left Heschl gyrus, and left insula and left parsorbitalis. Detailed network information can be found in Table 1 and Figure 2.

### 3.4. Association McGurk-Effect–WM Connectivity

#### 3.4.1. ADHD

**Figure 3:**
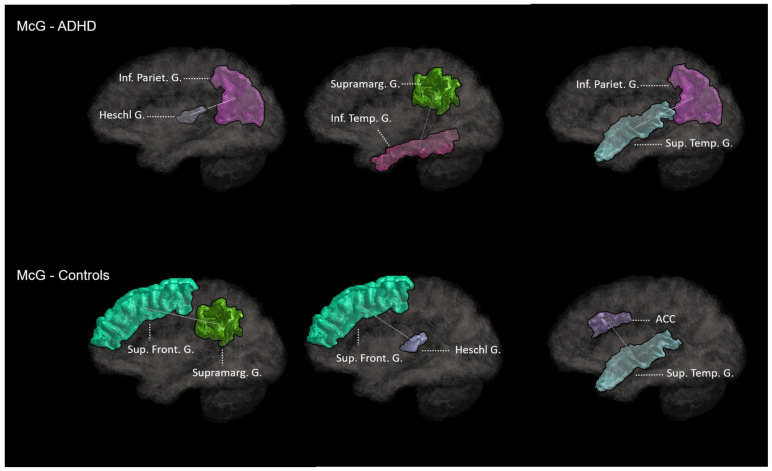
Brain–behavior relationship of integration with structural connectivity for ADHD (**top**) and controls (**bottom**); Abbreviations: Inf. Pariet. G., inferior parietal gyrus; G, gyrus; supramarg. G., supramarginal gyrus; sup. temp. G, superior temporal gyrus; sup. front. G, superior frontal gyrus; Acc, anterior cingulate cortex.

MCG scores correlated significantly with network connectivity between the right anteriorcingulate cortex (ACC, rostral part) and left anteriorcingulate cortex (caudal part), right superiortemporal gyrus (STG) and right inferiorparietal gyrus (IPG), left inferiortemporal gyrus (ITG) and left supramarginal gyrus (SMG), and right Heschl gyrus and right inferiorparietal gyrus (IPG). Detailed network information can be found in Table 1 and Figure 3.

#### 3.4.2. Controls

MCG-scores in healthy controls were significantly associated with network connectivity between the left caudate and left supramarginal gyrus (SMG), right thalamus and left supramarginal gyrus (SMG), left supramarginal gyrus and left superiorfrontal gyrus (SFG), right superiortemporal gyrus (STG) and right anterior cingulate cortex and right precuneus and left superior temporal sulcus (STS), and left Heschl gyrus and left superior frontal gyrus (SFG). Detailed network information can be found in Table 1 and Figure 3.

### 3.5. Graph Theory

Global efficiency and shortest path length yielded no significant results. Patients with ADHD showed higher nodal local efficiency for left isthmuscingulate, left/right lateral occipital cortex, left pericalcarine fissure, and right inferiorparietal gyrus. Degree centrality was elevated in ADHD for the left postcentral gyrus compared to controls. Further, patients showed a higher clustering coefficient in the right lateraloccipital cortex (see Table 2).

## 4. Discussion

To the best of our knowledge, this is the first study investigating the relationship between multisensory integration and structural connectivity in an adult ADHD sample compared to a healthy control group. Connectivity measures were calculated based on diffusion MRI-based structural connectome analyses and graph–theoretical approaches and correlated to fused MI performance. In ADHD, MI was significantly associated with network connectivity between Heschl gyrus and IPG along with connectivity within ACC and between IPG and SMG. MI in controls was significantly associated with network connectivity between thalamus and SMG, Heschl gyrus, and the precuneus, STG, and ACC. Overall, compared to controls, patients with ADHD elicited higher network integrity within temporal and occipital networks, while controls showed higher global connectivity in fronto-temporal, fronto-limbic, and fronto-insular networks. In children with ADHD, sensory-processing deficits have been reported across the senses, i.e., visual, auditory, touch, and smell [4,42,43]. Those sensory-processing deficits seem to ”grow out” toward an unimpaired sensory modulation during adolescence and adulthood, except from the auditory domain, where studies reported increasing issues over time [43]. As a consequence, adult ADHD may arise with auditory hypersensitivity, which may be rooted in an inhibition failure at early stimulus modulatory components [6]. In the current analysis, we found that Heschl gyrus had a stronger connectivity to STG in ADHD compared to controls. STG is also referred to as a parabelt region of the auditory cortex and has been reported to play a sensitive role in MI [44,45]. By integrating the visual and auditory information, STG receives input from the respective primary areas [45,46,47]. A higher connectivity from the primary auditory cortex to STG, as found in ADHD in our sample, could be interpreted in light of a higher cortical sensory weighting of auditory input in ADHD, hence explaining the auditory hypersensitivity. Interestingly, an auditory preference in audiovisual studies was also reported in healthy children [48]. Vision receives more cortical weight during learning of speech perception with age. In other words, this process of reweighting of sensory inputs could reflect structural–cortical maturation toward higher multisensory integration. Since ADHD is a neurodevelopmental disorder, with dysfunctional network integration (see discussion below), the elevated connectivity associated with auditory functions in our study could also reflect the deficient structural maturation process with potential consequences for MI behavior. In line with this interpretation, we found higher nodal local efficiency in the occipital cortex/pericalcarine fissure in ADHD. Those results could be an indicator of a higher cortical local visual processing, which could have consequences on cortical information transfer to other areas, e.g., sensory or polymodal areas. In healthy controls, we found sensory connections associated with MI in network connectivity between precuneus, a region that is also involved in bottom–up visual processing [49], and STS. Those cross-modal projections are known to play a crucial role in temporal aspects of MI [49]. This network connectivity was neither evident nor associated with MI in ADHD. Instead, in ADHD, MI was associated with network connectivity between temporo-parietal networks while controls seem to recruit more fronto-temporal networks in MI scenarios. Although the process of MI is far from being disentangled on the cortical level, one could distinguish between an early, bottom–up and late, top–down attentional control of MI [13,50,51]. While the bottom–up MI is rather localized within temporo-parietal regions, top–down underlies frontal-temporal/parietal regions. The latter has been found to be responsible to account for increasing perceptual conflict in uncertainty situations and prediction error processing [52]. In order to enhance stimuli saliency at the top–down level, modulatory influence from frontal areas to sensory areas and vice versa are necessary [48]. Based on the results of this study, we assume that this top–down modulatory influence is disrupted in ADHD, since the early integration is dominated by temporo-parietal connectivity. In contrast, controls do show sufficient MI, triggered through influential late top–down response enhancement, as we found MI-related connectivity between thalamus and SMG and between STG and ACC. To summarize, we assume an enhanced early bottom–up and a disrupted late top–down MI in ADHD that is moderated through an unequal cross-sensory weighting process in favor of the auditory sensory modality.

Furthermore, ADHD pathophysiology has been strongly associated to dysfunctional resting-state connectivity and regional brain abnormalities [53]. In particular, an important role in ADHD has been attributed to the DMN, since it is strongly correlated to the attention deficit [54]. Moreover, the evidences provided by structural connectivity studies increasingly point toward dysfunctional WM network integration in ADHD, further highlighting the possibility that inattention is caused by abnormal neuronal inter-regional communication. It has been found that inattention is accompanied with a higher nodal degree in the hippocampus, SMG, calcarine sulcus, and occipital cortex [15,55,56]. In our study, we also found a higher nodal degree in the lateraloccipital cortex along with higher connectivity between the entorhinal cortex and the cerebellum, a region also associated with the pathophysiology of ADHD [57]. Additionally, ADHD showed higher network communication between entorhinal cortex and insula. The insula is part of the salience network; it is associated with integration of sensory stimuli. Resting-state functional connectivity revealed a negative correlation of the insula with ability to integrate sensory stimuli in ADHD [12]. Additionally, dysfunctional network integration has been reported for precuneus and limbic regions, e.g., putamen [15,55,56]. To summarize, our findings are in line with ADHD-related dysfunctional network integration known from the literature.

## 5. Conclusions

We demonstrated that the ability to integrate top–down multisensory information in patients with ADHD is associated with bottom–up networks. Missing top–down network involvement for complex stimuli integration might represent the maturational cortical delay in ADHD that extends to adulthood with respect to sensory processing.

## Figures and Tables

**Table 1:** Network statistics for ADHD vs. controls and brain–behavior relationship.

Network	z-Value	*p*-Value
ADHD > Controls		
R entorhinal C.–R Insula	2.15	0.032
L entorhinal C.–R Cerebellum	2.05	0.045
L superior temp. G.–L Heschl G.	2.05	0.039
R Putamen–R Precuneus	1.95	0.033
L parsopercularis–R Precuneus	1.94	0.032
ADHD < Controls		
R anterior cing. C.–R superior temp. sulcus	2.73	0.018
R Cuneus–L lingual G.	2.67	0.048
R Insula–R parsorbitalis	2.6	0.033
L Accumbens–L paracentral G.	2.44	0.009
R paracentral G.–L Heschl G.	2.29	0.009
L Insula–L parsorbitalis	2.27	0.002
Super. parietal G.–Hippocampus	2.19	0.003
McG–ADHD		
R anterior cing. C. (rostral) –L anterior cing. C. (caudal)	2.2	0.033
R super temp. G.–R inferior parietal G.	2.19	0.031
L inferior temp. G.–L supramarginal G.	2.2	0.035
R Heschl G.–R inferior parietal G.	2.3	0.032
McG–Controls		
L Caudate–L supramarginal G.	2.29	0.044
R Thalamus–L supramarginal G.	2.28	0.042
L supramarginal G.–L superior front. G.	2.28	0.041
R superior temp. G.–R anterior cing. C.	2.08	0.044
L superior temp. S.–R Precuneus	2.07	0.043
L Heschl G.–L superior front. G.	2.06	0.042

Abbreviations: R, right; L, left; C., cortex; G., gyrus; cing., cingulate; temp., temporal; front., frontal; note: results are familywise error-corrected (FEW; *p* = 0.05).

**Table 2:** Graph–theoretic results where all results denote ADHD > controls.

Measure	t-Value	*p*-Value
Degree Centrality		
R postcentral G.	2.11	0.042
Nodal Local Efficiency		
L isthmuscingulate	2.19	0.034
L lateraloccipital C.	2.19	0.031
L pericalcarine Fissure	1.92	0.006
R inferiorparietal G.	2.16	0.033
R lateraloccipital C.	2.09	0.042
Clustering Coefficient		
R lateraloccipital C.	2.04	0.046

Abbreviations: R, right; L, left; C., cortex; G., gyrus; note: results are false discovery rate (FDR)-corrected (*p* = 0.005).

## Data Availability

The datasets are available upon request.
